# Tricyclic and tetracyclic antidepressants upregulate VMAT2 activity and rescue disease-causing VMAT2 variants

**DOI:** 10.1038/s41386-024-01914-2

**Published:** 2024-07-26

**Authors:** Xunan Wang, Ilias Marmouzi, Peter S. B. Finnie, Meghan L. Bucher, Yuanye Yan, Erin Q. Williams, Svein I. Støve, Tatiana V. Lipina, Amy J. Ramsey, Gary W. Miller, Ali Salahpour

**Affiliations:** 1 Department of Pharmacology and Toxicology, Temerty Faculty of Medicine, University of Toronto, Toronto, ON M5S 1A8 Canada; 2 Department of Environmental Health Sciences, Mailman School of Public Health, Columbia University, New York, NY 10032 USA; 3 Department of Biomedicine, University of Bergen, Jonas Lies vei 91, 5009 Bergen, Norway; 4 Neuro-SysMed, Department of Neurology, Haukeland University Hospital, 5021 Bergen, Norway; 5 K.G. Jebsen Center for Translational Research in Parkinson’s Disease, University of Bergen, 5020 Bergen, Norway; 6 Department of Physiology, Temerty Faculty of Medicine, University of Toronto, Toronto, ON M5S 1A8 Canada; 7 Department of Molecular Pharmacology and Therapeutics, Vagelos College of Physicians and Surgeons, Columbia University, New York, NY 10031 USA

**Keywords:** Pharmacology, Synaptic vesicle endocytosis, Depression, Target validation, Neurotransmitters

## Abstract

Vesicular monoamine transporter 2 (VMAT2) is an essential transporter that regulates brain monoamine transmission and is important for mood, cognition, motor activity, and stress regulation. However, VMAT2 remains underexplored as a pharmacological target. In this study, we report that tricyclic and tetracyclic antidepressants acutely inhibit, but persistently upregulate VMAT2 activity by promoting VMAT2 protein maturation. Importantly, the VMAT2 upregulation effect was greater in BE(2)-M17 cells that endogenously express VMAT2 as compared to a heterologous expression system (HEK293). The net sustained effect of tricyclics and tetracyclics is an upregulation of VMAT2 activity, despite their acute inhibitory effect. Furthermore, imipramine and mianserin, two representative compounds, also demonstrated rescue of nine VMAT2 variants that cause Brain Monoamine Vesicular Transport Disease (BMVTD). VMAT2 upregulation could be beneficial for disorders associated with reduced monoamine transmission, including mood disorders and BMVTD, a rare but often fatal condition caused by a lack of functional VMAT2. Our findings provide the first evidence that small molecules can upregulate VMAT2 and have potential therapeutic benefit for various neuropsychiatric conditions.

## Introduction

Monoamine regulation in the central nervous system (CNS) is important for cognition, motor control, mood, arousal, stress, and temperature regulation [1–3]. Altered monoamine transmission is hypothesized to be one of the underlying causes of depression and anxiety [4, 5]. Vesicular monoamine transporter 2 (VMAT2) is an important transporter for monoamine transmission within the CNS and plays a central role in synaptic vesicular packaging of monoamine neurotransmitters, which include serotonin, dopamine, norepinephrine, epinephrine, and histamine [6, 7]. Rare homozygous variants in the *SLC18A2* gene, which encodes the VMAT2 protein, result in brain monoamine vesicular transport disease (BMVTD) [8, 9]. Symptoms range from global developmental delay and hypotonia in mild cases to childhood lethality in severe cases [8, 9]. In addition, lower VMAT2 levels are associated with the risk of Parkinson’s disease and VMAT2 inhibition by reserpine induces depression in humans [10–15].

Since the 1950s, the monoamine system has been targeted pharmacologically for depression and mood disorders by monoamine oxidase inhibitors (MAOIs) and tricyclic antidepressants (TCAs) and since the 1980s by selective serotonin reuptake inhibitors (SSRIs) [16]. However, unlike plasma membrane transporters for serotonin (SERT) and norepinephrine (NET), the established targets for TCAs, VMAT2 has remained an underexplored pharmacological target for mood disorders and other indications [7, 16].

Our study aimed to identify compounds that could upregulate VMAT2 levels and activity since low VMAT2 levels are associated with BMVTD, Parkinson’s disease, and depression, and high VMAT2 levels are protective for Parkinson’s disease risk in humans and neurotoxicity in rodents [8–15, 17, 18]. We hypothesized that tricyclic antidepressants modulate VMAT2 activity since they are known to affect the monoamine transporters SERT and NET. We also explored the effect of tetracyclic antidepressants on VMAT2 functionality. Our results show that acute treatments with tricyclic and tetracyclic antidepressants inhibit VMAT2 activity, while sustained treatments upregulate VMAT2 protein levels and activity. Furthermore, our results show that these compounds can rescue the activity of nine BMVTD-causing missense VMAT2 variants that impair VMAT2 function in cells. Overall, our study is the first to report upregulation of VMAT2 protein and activity using clinically-effective tricyclic and tetracyclic antidepressants.

## Materials and methods

The suppliers of reagents are listed in Table S1.

### Plasmids

The full-length mouse and human *SLC18A2* cDNAs encoding VMAT2 were cloned into pcDNA3.1 vectors. A YFP-HA-DAT plasmid encoding WT human dopamine transporter (DAT) with a C-terminal EYFP tag and an HA-tag in the second extracellular loop (in pEYFP-C1 vector) was a gift from Sorkin Lab, University of Pittsburgh [19]. Constructs for human VMAT2 BMVTD variants were generated by PCR-based site-directed mutagenesis using primer pairs in Table S2 [20].

### Cell lines

Cell lines were maintained in a 37 °C incubator with 5% CO_2_. HEK293, HEK293T, and BE(2)-M17 cells were obtained from ATCC. The HEK-VMAT2 cell line was generated by stably transfecting the mouse VMAT2 cDNA expression construct in HEK293 cells. The HEK-mCherry-VMAT2 cells were generated previously [21]. The HEK-YFP-DAT stable cell line was generated previously [22]. The HEK-SERT stable cell line was a gift from Randy Blakely’s lab, Florida Atlantic University [23]. All cell lines were maintained in DMEM medium supplemented with 10% fetal bovine serum, 100 units/mL penicillin, and 100 μg/mL streptomycin. Stable cell lines were additionally supplemented with 0.5 mg/mL G418 Sulfate.

### FFN206 uptake assay

Detailed description of the FFN206 uptake assay was written in supplementary methods.

### Epifluorescence microscopy

Detailed description of the epifluorescence microscopy experiment was written in supplementary methods.

### Flow cytometry

BE(2)-M17 cells were seeded at 100,000 cells/well density in clear 24-well plates. On day 2, the growth medium was aspirated and 500 μL of 10 μM drug-containing medium (0.1% DMSO) or vehicle were added to each well and incubated for 18 h. On day 3, the drug-containing medium was aspirated and washed 3 times with 500 μL warm medium, each with 20 min incubation. After the wash, 450 μL medium containing 100 μM reserpine (to define VMAT2-independent uptake) diluted in 1% DMSO or vehicle were added to each well and incubated for 30 min. After incubation, 50 μL of 50 μM FFN206 diluted in PBS was added to each well and incubated for 1 h. The FFN206 uptake was terminated by a 1 mL warm PBS wash and then trypsinized with 200 μL trypsin+EDTA (0.25%). The cells were then resuspended in 400 μL flow cytometry buffer (25 mM HEPES, pH7.0, 1% bovine serum albumin, 1 mM EDTA, in 1X PBS) and kept on ice. Flow cytometry was performed under operator assistance at the University of Toronto Flow Cytometry Facility using BD LSR Fortessa™ X-20 flow cytometer with a 355 nm laser and a 450/50 nm filter. The flow cytometry data was analyzed with the FlowJo software. Example gating can be found in Fig. S1. For each overnight treatment with compound, acute treatment with 100 μM reserpine on day 3 was used to define VMAT2-independent uptake and the signal was subtracted from each drug-treated condition during data analysis. The mean fluorescence intensity (MFI) of the FFN+ population was quantified and normalized to cells that were treated with vehicle.

### Western blot

HEK293, HEK-VMAT2, HEK-YFP-DAT, and HEK-SERT cells were seeded at 5,000,000 cells/dish density in 10 cm tissue culture dishes. The next day, the growth medium was aspirated and 10 mL of medium (0.1% DMSO) containing 50 μM or 100 μM imipramine or mianserin or vehicle were added to the cells and incubated for 18 h. On the collection day, cells were washed once with cold PBS, then harvested with 2 mL Tris-EDTA buffer (25 mM Tris-HCl, 1 mM EDTA, pH 7.4) containing freshly-added protease/phosphatase inhibitors. The cells were mechanically lysed on ice by Polytron (medium setting, 5 sec on, 5 sec off, two cycles), and centrifuged at 800 x *g* for 10 min at 4 °C to pellet the nuclear fraction. The resulting supernatant was transferred into a new set of tubes and centrifuged again at 29,097 × *g* for 40 min at 4 °C. The supernatant was discarded and the pellet containing membrane proteins was resuspended with Tris-EDTA buffer with inhibitors and 0.2% SDS by vortexing until the pellet was dissolved. The protein concentration was determined using Pierce™ BCA Protein Assay Kits following the manufacturer’s instructions.

For the deglycosylation experiment, 22 μg of vehicle-treated VMAT2 protein lysate were digested with endoglycosidase H (Endo H), peptide:N-glycosidase F (PNGase F), or reaction buffer following manufacturer’s non-denaturing condition protocol for 24 h.

Samples were heated at 55 °C for 10 min before proteins were loaded onto SDS-polyacrylamide gels (NuPAGE™ 4–12% Bis-Tris Mini Protein Gel). Electrophoretic resolution was done in MOPS-SDS running buffer (50 mM MOPS, 50 mM Tris, 0.1% SDS, 1 mM EDTA). The proteins were transferred onto a 0.45 nm PVDF membrane and stained using Revert™ 700 Total Protein Stain. The resolved proteins were visualized at 700 nm on an Odyssey® M imaging system (LI-COR, Inc.). The membrane was blocked with 3% non-fat milk in TBST (19 mM Tris, 137 mM NaCl, 2.7 mM KCl, 0.1% Tween 20) for 1 h and then incubated in primary antibodies diluted in 3% non-fat milk in TBST. For the VMAT2 blot, the membrane was incubated with rabbit serum (in 50% glycerol) containing anti-mVMAT2 antibody against C-terminal peptide (TQNNVQPYPVGDDEESESD) at 1:3000 dilution overnight at 4 °C [24]. For the YFP-DAT blot, the membrane was incubated with rabbit-anti-GFP antibody (A11122) at 1:1500 dilution for 2 h at room temperature. For the SERT blot, the membrane was incubated with rabbit anti-SERT antibody (A104336, Antibodies.com) at 1:1000 dilution overnight at 4 °C. IRDye® 800CW Goat anti-Rabbit IgG secondary antibody at 1:7000 dilution in 3% non-fat milk in TBST was used for all blots. The blots were visualized under 700 nm and 800 nm channels on the Odyssey® M imaging system (LI-COR, Inc.) and quantified using ImageJ and Empiria Studio.

### Transient transfection of BMVTD variants

On day 1, HEK293T cells were seeded at 50,000 cells/well density in PDL-precoated black-bottomed 96-well plates. Four hours after seeding, 50 ng of human VMAT2 (WT or BMVTD variant) plasmid and 50 ng of pcDNA3.1 empty plasmid were co-transfected into each well using Lipofectamine^TM^ 3000, following the manufacturer’s protocol for 96-well plate experiments. Twenty-two hours post transfection, medium was aspirated and either drug (desired concentration of imipramine or mianserin)-containing media (0.05% DMSO) or vehicle (0.05% DMSO) was added to the cells. Eighteen hours after, the FFN206 uptake assay was performed as described in supplementary materials for upregulation assay. Western blot was conducted 20–24 h post-transfection as described in the methods for western blot, using rabbit serum (in 50% glycerol) containing anti-hVMAT2 antibody against C-terminal peptide (TQNNVQPYPVGDDEESESD) at 1:3000 dilution in 3% non-fat milk in TBST [25].

### RT-qPCR

HEK-VMAT2, HEK-YFP-DAT, HEK-SERT, and BE(2)-M17 cells were seeded in 6-well tissue culture plates at 1,000,000 cells/well density. The next day, the growth medium was aspirated and 10 mL of medium (0.1% DMSO) containing 100 μM (for HEK-VMAT2 and HEK-YFP-DAT cells) or 10 μM (for BE(2)-M17 cells) imipramine or mianserin or vehicle were added to the cells and incubated for 18 h. On the third day, medium was aspirated and the cells were directly collected in 1 mL/well of Trizol for RNA isolation [26]. RNA was reverse-transcribed into cDNA using the SuperScript™ IV VILO™ Master Mix with ezDNase™ Enzyme. Quantitative RT-qPCR was performed using PowerUp™ SYBR™ Green Master Mix and QuantStudio 3 Real-Time PCR System (ThermoFisher Scientific). Primer pairs used for quantitative PCR and primer efficiencies are listed in Table S3. The relative fold of gene expression was calculated as 2^-(∆∆Ct) normalized to GAPDH levels.

## Results

### Acute treatment with tricyclics or tetracyclics inhibits VMAT2 activity

**Fig. 1:**
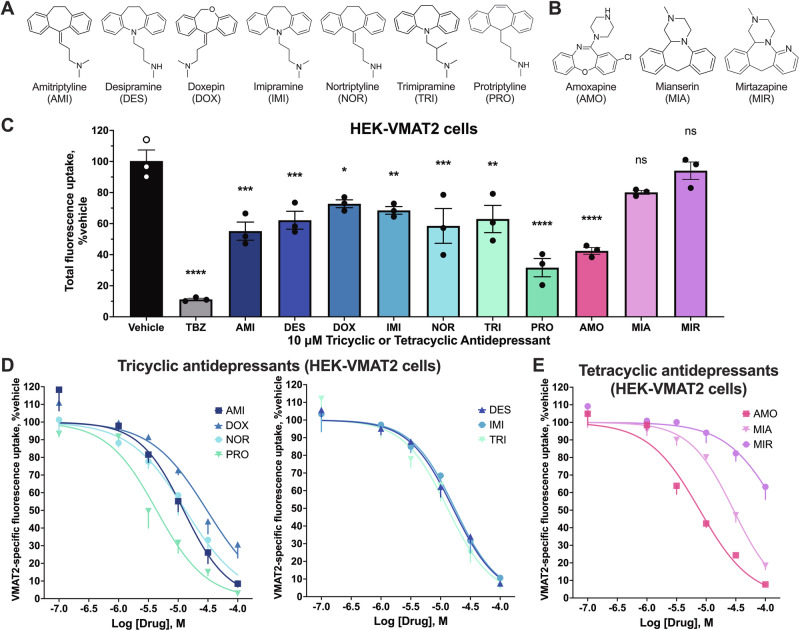
Acute effect of tricyclics and tetracyclics on VMAT2 activity. **A** Chemical structures of tricyclics tested. **B** Chemical structures of tetracyclics tested. **C** Effect of 30-min incubation with 10 μM tricyclics or tetracyclics on VMAT2 activity in HEK-VMAT2 cells. One-way ANOVA was performed followed by Dunnett’s test comparing each treatment against the vehicle (*n* = 3). *P* > 0.05 (ns), ≤0.05 (*), ≤0.01 (**), ≤0.001 (***), ≤0.0001 (****). **D**, **E** Concentration-response curve for inhibition by tricyclics and tetracyclics of VMAT2 activity in HEK-VMAT2 cells (*n* = 3). For all concentrations, non-specific uptake (FFN206 signal of vehicle under 100 μM TBZ inhibition) was subtracted in data analysis. All treatments were normalized to vehicle (100%).

FFN206 is a fluorescent substrate of VMAT2 that gets transported into VMAT2-containing intracellular compartments such as vesicles [21, 27]. We detected colocalization of VMAT2 and FFN206 using epifluorescence microscopy (Fig. S2) and used FFN206 uptake to measure VMAT2 activity in HEK cells stably expressing mouse VMAT2 (HEK-VMAT2). We tested seven tricyclics and three tetracyclics that are approved for clinical use in humans (Fig. 1A, B). The acute effect of the drugs on VMAT2 activity was first assessed by pre-incubating HEK-VMAT2 cells with test compounds for thirty minutes followed by FFN206 co-incubation. Thirty-minute treatment with 10 μM of all tested tricyclics or the tetracyclic amoxapine significantly inhibited VMAT2 activity (Fig. 1C). Control experiments on HEK293 cells not expressing VMAT2 showed no difference in FFN206 uptake after 30-min tricyclic or tetracyclic treatment (Fig. S3). Next, we assessed the potency of the tricyclics and tetracyclics using dose response experiments. All drugs demonstrated comparable dose-dependent inhibition of VMAT2 with low-to-mid micromolar potency (IC_50_), except for mirtazapine (Fig. 1D, E). Protriptyline (IC_50_ = 4.35 μM) and amoxapine (IC_50_ = 7.79 μM) were the most potent tricyclic and tetracyclic, respectively (Table 1). Compared to tetrabenazine (TBZ) (Fig. S4), the prototypical VMAT2 inhibitor, tricyclics and tetracyclics inhibited VMAT2 with a lower potency (higher IC_50_) but similar maximum inhibition (I_max_) (Table 1).

**Table 1:** Comparison of VMAT2 inhibition and upregulation effects by tricyclics and tetracyclics^a^.

		Inhibition (30 min)			Upregulation (18 h)			
		IC_50_^b^ (95% CI) (μM)	I_max_^c^, % vehicle (mean ± SEM)	Hill Slope^b^	EC_50_^d^ (95% CI) (μM)	E_max_, % vehicle (mean ± SEM)	E_max_ concentration (μM)	Hill slope^d^
Tricyclics	AMI	12.36 (8.45–18.17)	8.47 ± 1.88	–1.173	^e^	237.26 ± 7.63	31.62	1.728
	DES	16.16 (13.37–19.54)	7.51 ± 1.28	–1.176	17.1 (6.776–?)^f^	177.59 ± 5.96	56.23	1.530
	DOX	30.5 (22.00–43.65)	30.72 ± 7.66	–0.922	42.41 (26.73–297)	243.49 ± 19.69	100	1.494
	IMI	17.31 (13.45–22.28)	10.67 ± 2.24	–1.195	29.86 (17.58–?) ^f^	245.36 ± 15.06	100	1.505
	NOR	13.26 (9.27–18.90)	8.73 ± 1.11	–0.920	^e^	207.90 ± 18.90	31.62	0.915
	TRI	13.07 (9.43–18.02)	5.87 ± 1.33	–1.173	31.1 (20.5–1277)	194.63 ± 22.32	100	1.939
	PRO	4.35 (3.19–5.94)	2.93 ± 0.24	–1.042	^e^	223.92 ± 11.02	31.62	0.777
Tetracyclics	AMO	7.79 (5.94–10.27)	7.73 ± 1.23	–1.002	18.46 (15.38–22.51)	179.83 ± 18.89	31.62	4.376
	MIA	29.04 (23.89–35.39)	18.41 ± 2.14	–1.172	29.86 (20.44–375)	223.48 ± 8.24	100	1.871
	MIR	169.8 (109.9–419.9)	63.27 ± 7.1	–0.978	35.1 (21.64–?) ^f^	175.11 ± 7.95	100	2.486
Control	TBZ	0.037 ^g^ (0.028–0.049)	9.51 ± 0.67	–0.598				

^a^All VMAT2 activity data showed in the table was after subtraction of non-specific uptake (FFN206 signal of vehicle under 100 μM TBZ inhibition) and normalized to vehicle treatment (100%).

^b^The IC_50_ and Hill Slope were generated from data on Fig. 1D, E, curve fitted using GraphPad PRISM built-in analysis nonlinear regression - “log(inhibitor) vs normalized response – variable slope” with a top constraint of 100%.

^c^I_max_ represents inhibition of VMAT2 activity by the 100 μM compounds as shown in Fig. 1D, E.

^d^The EC_50_ and Hill Slope were obtained using the data on Fig. 2B, C curve fitted using GraphPad PRISM built-in analysis nonlinear regression - “Sigmoidal, 4PL, X is log (concentration)” with a bottom constraint of 100%.

^e^EC50 cannot be estimated accurately because of non-saturated dose-response curves.

^f^GraphPad PRISM cannot give an estimate for the upper bound of 95% confidence interval.

^g^Estimated using the complete dose-response curve in Fig. S4.

### Sustained treatment with tricyclics or tetracyclics upregulates VMAT2 activity

**Fig. 2:**
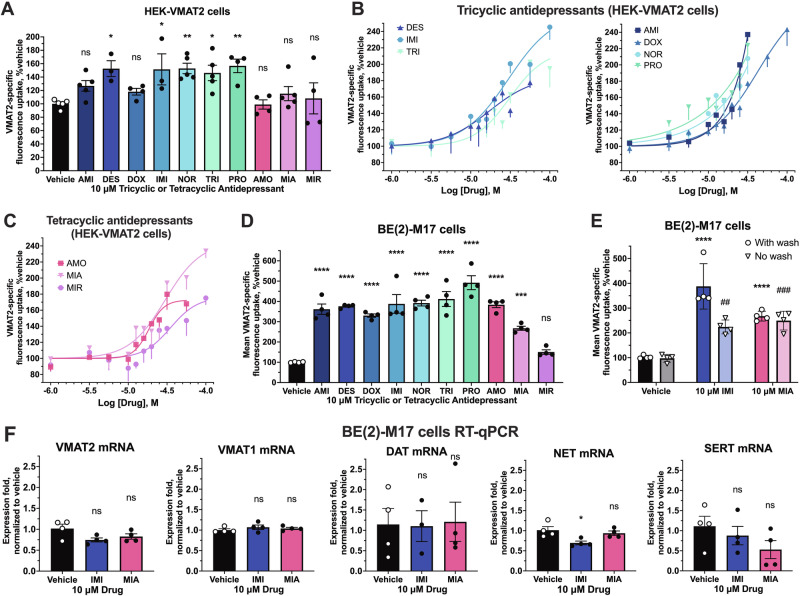
Sustained effect of tricyclics and tetracyclics on VMAT2 activity. **A** Effect on VMAT2 activity of 18-h incubation with 10 μM tricyclics or tetracyclics followed by washout in HEK-VMAT2 cells. One-way ANOVA was performed followed by Dunnett’s test comparing each treatment against the vehicle (*n* = 3–5). *P* > 0.05 (ns), ≤0.05 (*), ≤0.01 (**), ≤0.001 (***), ≤0.0001 (****). VMAT2-specific uptake was obtained by subtracting the fluorescence signal of each condition under 100 μM TBZ inhibition. **B**, **C** Concentration-response curve of VMAT2 activity after 18-h incubation with each tricyclic or tetracyclic at increasing concentrations, followed by washout in HEK-VMAT2 cells (*n* = 3–6). For all concentrations, non-specific uptake (FFN206 signal of vehicle under 100 μM TBZ inhibition) was subtracted in data analysis. All treatments were normalized to vehicle (100%). **D** Effect on endogenous VMAT2 activity of 18-h incubation with each tricyclic or tetracyclic at 10 μM, followed by washout in BE(2)-M17 cells (*n* = 4). One-way ANOVA was performed followed by Dunnett’s test comparing each treatment against the vehicle. **E** Effect on endogenous VMAT2 activity of 18-h incubation with 10 μM imipramine and mianserin with or without washout in BE(2)-M17 cells (*n* = 4). Two-way ANOVA was performed followed by Dunnett’s test comparing between drug and vehicle for each wash condition. *P* > 0.05 (ns), ≤0.05 (* or ^#^), ≤ 0.01 (** or ^##^), ≤ 0.001 (*** or ^###^), ≤ 0.0001 (**** or ^####^). For each independent experiment, all treatments were normalized to vehicle (100%). For (**D**, **E**), VMAT2-independent uptake of FFN206 was defined by inhibition with 100 μM reserpine after 18-h incubation with test compounds and was subtracted in data analysis. All treatments were normalized to vehicle (100%). **F** RT-qPCR of endogenous monoamine transporters mRNA in BE(2)-M17 cells after 18-h treatment with 10 μM imipramine or mianserin (*n* = 4).

With the knowledge that some inhibitors could also act as pharmacological chaperones of the given target and increase its protein maturation and levels, we then tested the sustained effect of each tricyclic and tetracyclic on VMAT2 activity after an 18-h treatment [22]. To prevent the presence of inhibitors masking a VMAT2 upregulation effect (measured by FFN206 uptake activity), we incorporated three 20-min washes to remove any remaining drug after the treatment. In HEK-VMAT2 cells, 18-h treatment with the majority of evaluated tricyclics, including desipramine, imipramine, nortriptyline, trimipramine, and protriptyline, caused significant upregulation of VMAT2 activity at 10 μM concentration (Fig. 2A). In contrast, at 10 μM concentration, no sustained tetracyclics treatment significantly upregulated VMAT2 activity (Fig. 2A). We next conducted dose-response curves in which all tricyclics and tetracyclics demonstrated a dose-dependent upregulation of VMAT2 with mid-micromolar to low-millimolar potency (EC_50_) (Fig. 2B, C). Desipramine (EC_50_ = 17.1 μM) and amoxapine (EC_50_ = 18.46 μM) were the most potent upregulators among tricyclics and tetracyclics, respectively (Table 1). Sustained treatment with 100 μM imipramine and mianserin resulted in the highest VMAT2 activity upregulation of their class at an E_max_ of 245.36% and 223.48%, respectively (Table 1). Known VMAT2 inhibitors TBZ and haloperidol were also assessed for 18-h treatment, no upregulation of VMAT2 activity was observed (Fig. S5). To assess whether VMAT2 upregulation is only seen in an over-expression system, we carried out sustained treatment experiments on BE(2)-M17 cells, which endogenously express low levels of human VMAT2. We used flow cytometry to measure uptake of FFN206 by BE(2)-M17 cells because the endogenous levels of VMAT2 were below the detection limit of the plate reader used with HEK cells. Reproducible and larger-fold VMAT2 upregulation was observed in BE(2)-M17 cells after sustained treatment with tricyclics and tetracyclics. At 10 μM concentrations, all tricyclics and tetracyclics except mirtazapine demonstrated significant upregulation of VMAT2 activity ranging from 152% to 498% of vehicle (Fig. 2D).

To delve into the upregulation effect, we selected imipramine and mianserin, the compounds with the highest E_max_ in upregulation effect amongst their class, as representative compounds from the tricyclics and tetracyclics, respectively (Table 1). Because a larger upregulation effect of tricyclics and tetracyclics was seen in BE(2)-M17 cells compared to HEK-VMAT2 cells, we hypothesized that, under sustained treatment, the upregulation effect would outweigh the inhibitory effect. We therefore tested the net effect of sustained imipramine or mianserin treatment with and without drug washout. As shown in Fig. 2E, sustained treatment without washout still lead to a significant upregulation of endogenous VMAT2 activity in BE(2)-M17 cells.

We next investigated whether the upregulation of VMAT2 activity by tricyclics or tetracyclics could be explained by upregulation of gene expression of VMAT2 or related monoamine transporters (VMAT1, DAT, NET, and SERT) in BE(2)-M17 cells, which express these transcripts endogenously. With 10 μM imipramine or mianserin treatment, despite a significant upregulation in activity was observed, no upregulation in any monoamine transporter mRNA, including VMAT2 was detected (Fig. 2F).

### Tricyclics and tetracyclics upregulate VMAT2 by promoting VMAT2 protein maturation

**Fig. 3:**
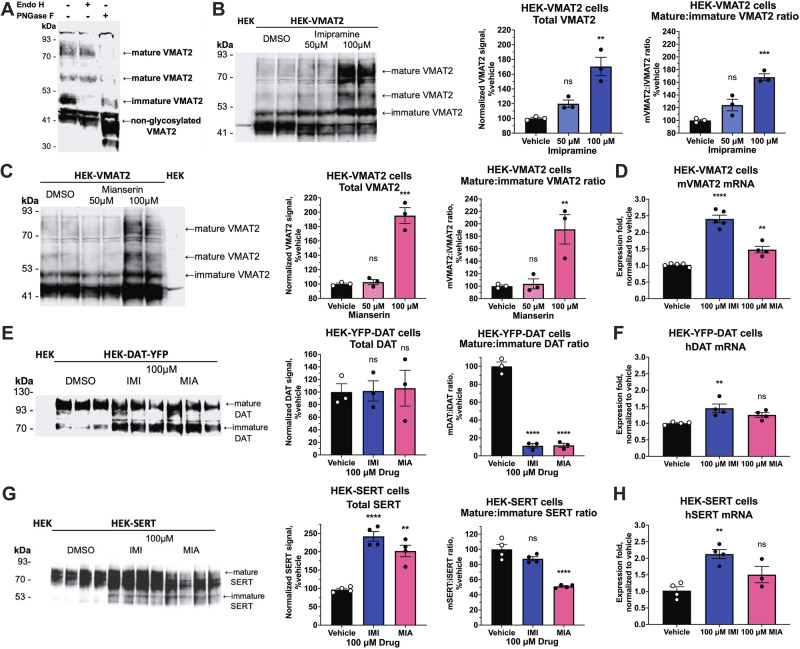
Sustained effect of tricyclics and tetracyclics on VMAT2 protein and mRNA. **A** Western blot of endoglycosidase H (EndoH) and peptide:N-glycosidase F (PNGase F) digested VMAT2 protein revealing different trafficking stages of the VMAT2 protein. **B**, **C** Representative western blot of 18-h imipramine or mianserin incubation on VMAT2 protein in HEK-VMAT2 cells (duplicate lanes of *n* = 1 displayed) and blot quantifications (*n* = 3). **E** Western blot for DAT protein after 18-h 100 μM imipramine or mianserin incubation in HEK-YFP-DAT cells (*n* = 3, each lane represents an independent experiment) and blot quantifications (*n* = 3). **G** Western blot for SERT protein after 18-h 100 μM imipramine or mianserin incubation in HEK-SERT cells (*n* = 4, each lane represents an independent experiment) and blot quantifications (*n* = 4). **D**, **F**, **H** RT-qPCR of mVMAT2, hDAT, hSERT in HEK293 cells following 18-h treatment with 100 μM imipramine or mianserin (*n* = 4–5). For all bar graphs, one-way ANOVA was performed followed by Dunnett’s test comparing each treatment against the vehicle. *P* > 0.05 (ns), ≤0.05 (*), ≤0.01 (**), ≤0.001 (***), ≤0.0001 (****). Primer efficiencies were assessed and reported in Supplementary Table S3.

We and others have observed that some transporter ligands can act as pharmacological chaperones that increase the ratio of mature:immature transporter [22, 28–31]. Therefore, we investigated whether drug treatment affected the process of VMAT2 protein maturation as a potential mechanism explaining the upregulation of VMAT2 activity by tricyclics and tetracyclics. As expected for a glycosylated transmembrane protein, antibodies against VMAT2 revealed proteins with molecular weights of 41, 50, 60, and 80 kDa in HEK-VMAT2 cells (Fig. 3A, S6). We incubated membrane protein a with deglycolysating enzymes to differentiate the immature and fully glycosylated isoforms of VMAT2. Deglycosylation by endoglycosidase H (Endo H) revealed that the 50 kDa band, which was sensitive to Endo H digestion, represents the immature VMAT2 located at the endoplasmic reticulum (ER) [22] (Fig. 3A). Deglycosylation by peptide:N-glycosidase F (PNGase F) revealed that both 60 kDa and 80 kDa bands, which were insensitive to Endo H digestion but sensitive to PNGase F digestion, represent the mature VMAT2 protein (Fig. 3A).

We next treated the HEK-VMAT2 cell line with imipramine and mianserin and determined the ratio between mature VMAT2 and immature VMAT2, where a higher ratio represents a greater extent of VMAT2 maturation. Western blot analysis of cells treated for 18-h with 100 μM imipramine or mianserin both showed a significant increase in total VMAT2 protein and mature:immature VMAT2 ratio (Fig. 3B, C, S7). The increase in mature:immature VMAT2 ratio suggests that imipramine and mianserin promote VMAT2 maturation. We were unable to assess the effects of compounds on VMAT2 protein levels in BE(2)-M17 cells because the VMAT2 protein levels in these cells were below the western blot detection threshold (data not shown).

To examine the selectivity of these compounds, we assessed the effect of imipramine and mianserin on DAT (*SLC6A3*) and SERT (*SLC6A4*) by western blot of stably transfected HEK-293 cells. Like VMAT2, mature DAT and SERT proteins are glycosylated and is detected by western blot in its mature (110 kDa and 80 kDa, respectively) and immature (70 kDa and 53 kDa, respectively) state (Fig. 3E, G) [22]. Tricyclics and tetracyclics are not known to be inhibitors of DAT, contrary to VMAT2 protein. 18-h treatment with 100 μM imipramine or mianserin did not affect total DAT protein and significantly decreased the mature:immature DAT ratio (Fig. 3E, S8). It is however known that tricyclics and tetracyclics act as inhibitors of SERT and an 18-h treatment with 100 μM imipramine or mianserin significantly increased total SERT protein but decreased the mature:immature SERT ratio for the mianserin treated samples (Fig. 3G, S9). Imipramine treatment elevated mRNA expression of VMAT2, DAT, and SERT in HEK293 cells, nevertheless, its effect on VMAT2, DAT, and SERT protein levels are different with increases seen for total VMAT2 and SERT and no effect on total DAT protein (Fig. 3D, F, H).

### Imipramine and mianserin rescue the activity of missense VMAT2 BMVTD variants

**Fig. 4:**
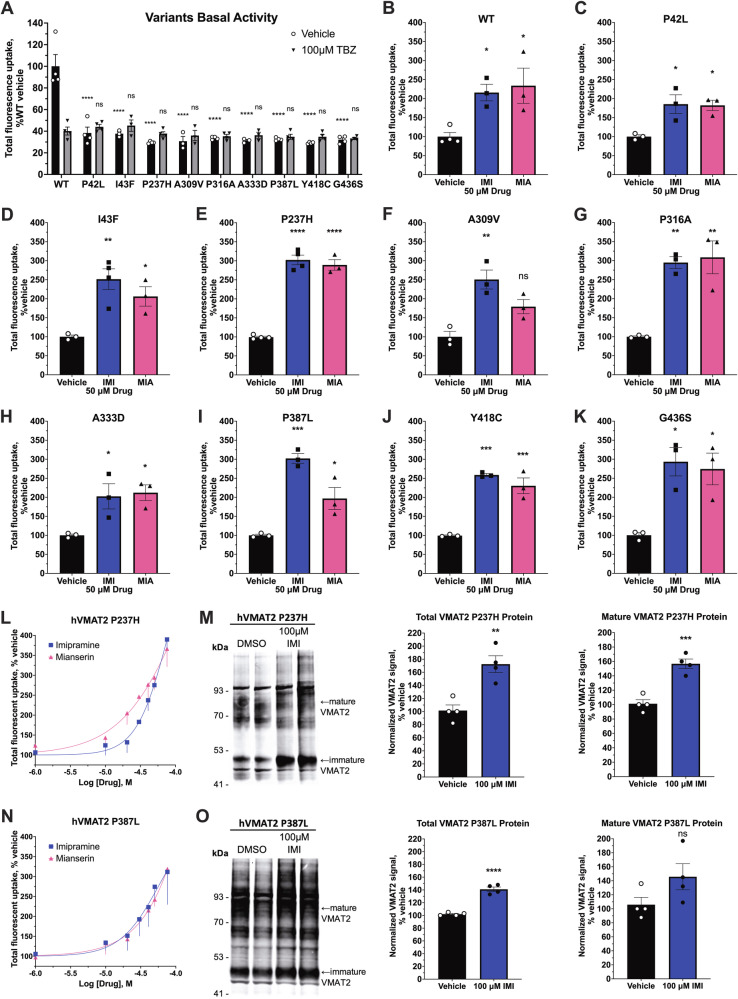
Sustained effect of imipramine and mianserin on the activity of VMAT2 BVMTD variants. **A** Basal VMAT2 activity of transiently transfected BVMTD variants in HEK293T cells (*n* = 5–8). Two-way ANOVA was performed followed by Dunnett’s test comparing each variant against WT for both vehicle and 100 μM TBZ conditions. **B–K** Transiently transfected VMAT2 WT and variant activity after 18-h treatment with 50 μM imipramine or mianserin (*n* = 3–4). **L**, **N** Concentration-response curve of VMAT2 P237H and P387L variants following 18-h treatment with increasing concentration of imipramine or mianserin in HEK293T cells transiently transfected with VMAT2 variants (*n* = 3). **M**, **O** Representative western blot (*n* = 2 were displayed) of human VMAT2 P237H and P387L protein after 18–20 h incubation with 100 μM imipramine in HEK293T cells transiently transfected with VMAT2 variants and blot quantifications (*n* = 4). For (**B–K**, **M–O**), one-way ANOVA was performed followed by Dunnett’s test comparing each treatment against the vehicle. *P* > 0.05 (ns), ≤0.05 (*), ≤0.01 (**), ≤0.001 (***), ≤0.0001 (****). For all VMAT2 variants FFN206 uptake experiments, total FFN206 fluorescence was displayed. VMAT2-independent uptake could not be measured and subtracted from the total uptake because the disease variants impaired TBZ inhibition. All treatments were normalized to vehicle (100%).

To explore potential clinical applications of VMAT2 upregulation by tricyclics and tetracyclics, we tested their effects on nine disease-causing, missense VMAT2 variants that were recently reported [8, 32]. All nine missense variant showed impaired VMAT2 uptake activity when studied in transiently-transfected HEK293T cells (Fig. 4A). Notably, TBZ inhibition in all variants were impaired. We then tested the effect of 18-h treatment with 50 μM imipramine or mianserin on all variants. Both imipramine and mianserin caused a significant upregulation of activity of all VMAT2 variants (Fig. 4B–K). The upregulation effect ranged from 179 % to 321 % of vehicle treatment. For most variants, the effect on VMAT2 activity was greater with imipramine than mianserin. We conducted dose-response uptake and western blot experiments with the two most recurrent variants, P237H and P387L. Similar to our observations on WT VMAT2, 18-h treatment with imipramine and mianserin showed a dose-dependent upregulation of variant VMAT2 activity (Fig. 4L, N). Furthermore, overnight treatment with imipramine significantly increased total and mature protein of P237H and P387L VMAT2 variants (Fig. 4M, O, S10–11).

## Discussion

We report that VMAT2 is a novel pharmacological target for several clinically-relevant tricyclic and tetracyclic antidepressants. Tricyclics and tetracyclics are hydrophobic compounds (imipramine logP = 4.8, mianserin logP = 3.4, PubChem) that act in the central nervous system. Hydrophobic compounds are able to permeate cell membrane and blood-brain barrier freely by passive diffusion [33]. Therefore, tricyclis and tetracyclics are also able to access intracellular targets such as VMAT2. With an apparent class effect, these antidepressants exhibit two modes of VMAT2 modulation: inhibition of VMAT2 activity with acute treatment, and upregulation of VMAT2 activity with sustained treatment. The mechanism of inhibition could be explained by a direct interaction between the drug and the target, causing inhibition of VMAT2 transporter activity. The mechanism of upregulation includes at least in part an effect on protein level and maturation. In BE(2)-M17 cells that endogenously express VMAT2 and related monoamine transporter transcripts, a drug concentration that result in 390% and 270% upregulation of VMAT2 activity by imipramine and mianserin showed no upregulation in VMAT2 mRNA (Fig. 2D, F). This examination of monoamine transporter gene expression suggests that drug effects of VMAT2 activity upregulation are not through transcriptional regulation.

In stable-transfected HEK293 cells, the total VMAT2 protein levels increased along with an increase in the mature:immature VMAT2 ratio, suggesting that tricyclics and tetracyclics promote VMAT2 protein maturation (Fig. 3B-C). This phenomenon of inhibitors promoting transporter maturation has previously been reported for ibogaine, bupropion, and their analogs on DAT, and ibogaine and its analogs on SERT [22, 28–31, 34]. Ibogaine and ibogaine analogs were proposed to act as pharmacological chaperones that bind to the inward-facing conformation of DAT and SERT and stabilize the folding of the transporters. Through stabilization of protein folding, pharmacological chaperones promote maturation of the protein and have an even more substantial effect on folding-deficient disease variants [22, 28, 31]. Since the effects of tricyclics and tetracyclics on VMAT2 upregulation and protein maturation were similar to those of pharmacological chaperones for DAT and SERT, we hypothesize that tricyclics and tetracyclics act as pharmacological chaperones of VMAT2. More investigation is needed to understand the precise mechanism by which tricyclics and tetracyclics promote VMAT2 protein maturation.

Pharmacologically upregulating VMAT2 protein and activity have potential important clinical implications. It is likely that VMAT2 upregulation is beneficial for mood disorders. Reserpine, an irreversible VMAT1 and VMAT2 inhibitor, was initially used as an anti-hypertensive drug [35]. However, numerous studies have reported depressive mood as an adverse effect of reserpine [14, 15]. The depressive effect of reserpine-induced monoamine depletion was early evidence pointing toward the role of monoamines in depression [4, 36]. Although the current understanding of the pathophysiological mechanisms of mood disorders and depression has changed to include monoamine deficiency, impaired neurogenesis, inflammation, and genetics, VMAT2 inhibition by reserpine remains a valid model of depression in preclinical animal models [37, 38]. Reciprocally, mice with genetically elevated VMAT2 level have higher levels of striatal dopamine content and are more resistant to anxiety-like and depressive-like behaviors [39]. Based on human and animal studies, VMAT2 expression levels and activity correlate with depressive mood, and thus upregulation of VMAT2 levels and activity could be explored for potential antidepressant effects.

The tricyclic and tetracyclics used in our study are well known to have antidepressant effects in humans, however it is unclear to what extent VMAT2 modulation explains their therapeutic effect. An argument against this possibility is the drugs’ mid-micromolar potency for VMAT2 upregulation. However, small molecules that promote VMAT2 maturation would upregulate VMAT2 levels and potentially enhance monoamine transmission. We hypothesize that more potent VMAT2 upregulators could potentially lead to the discovery of a novel class of antidepressants.

Upregulation of VMAT2 could also be beneficial for Parkinson’s disease. A genetic study has found that alleles resulting in VMAT2 overexpression (rs363371 and rs363324) are associated with a lower risk of developing Parkinson’s disease in an Italian population [11]. A study with the Chinese Han population has replicated the result for the rs363371 allele in males, but not the other allele [12]. Another human study has reported gain-of-function VMAT2 haplotypes that result in overexpression of VMAT2 are associated with lower risk of developing Parkinson’s disease in females [13]. Since genetic studies suggest that increased VMAT2 expression is associated with a protective effect for Parkinson’s disease, pharmacological upregulation of VMAT2 levels may also be protective.

Lastly, the rare genetic disease BMVTD, caused by homozygous mutations in the gene coding for VMAT2, could potentially be treated with VMAT2 upregulating small molecules. In our study, we found that all nine of the missense variants that we tested had VMAT2 functional impairment at baseline. We discovered that imipramine and mianserin upregulated the activity of all variants by 2- to 3-fold compared to vehicle treatment. Most importantly, the two most common recurring variants, P237H and P387L, showed ~300% upregulation of activity by imipramine compared to their baseline (Fig. 4E, I). P237H is the most prevalent and most severe BMVTD variant, which has a 53% patient mortality before 13 years of age and currently has no effective treatment options [8]. Our preliminary results in cells are promising as imipramine is FDA-approved for human use, and therefore could be tested for treating BMVTD patients in clinical trials [40]. However, with imipramine’s current potency for upregulating VMAT2 activity (EC_50_ = 29.86 μM), a therapeutic concentration is unlikely to be reached physiologically. Nevertheless, our study demonstrated that upregulating VMAT2 activity of disease variants with small molecule is possible and provided imipramine and mianserin as two small molecule framework for further chemical optimization of small molecule therapeutics for this rare disease indication. For a rare disease with limited treatment options like BMVTD, upregulation of VMAT2 activity by small molecules could potentially be a promising treatment for these BMVTD patients.

In conclusion, our study is the first to report that clinically-relevant tricyclic and tetracyclic antidepressants inhibit VMAT2 activity with acute treatment but upregulate VMAT2 activity with sustained treatment by promoting VMAT2 protein maturation. The upregulation effect was greater in the endogenous BE(2)-M17 cells than in the HEK cells. However, HEK cells were useful to detect the drugs’ effects on protein maturation using western blot to identify glycosylated protein forms. Imipramine and mianserin, representative tricyclic and tetracyclic, rescued the activity of fatal disease-causing variants of VMAT2.

A limitation of our study is that our findings are restricted to cell systems, therefore further investigation in vivo is necessary to ascertain whether tricyclics or tetracyclics also upregulate VMAT2 in vivo. Tricyclic and tetracyclic antidepressants have long been used in the clinic for the treatment of depression in humans, thus studies involving positron emission tomography (PET) imaging could be conducted to investigate if the VMAT2 protein upregulation phenomenon could be observed in patients who are taking tricyclic or tetracyclic antidepressants. In addition, our study identified imipramine and mianserin as two promising small molecule framework to start small molecule drug discovery for the fatal disease BMVTD.

## Supplementary information

Supplementary Materials

## Data Availability

Data will be available upon request to Dr. Ali Salahpour (ali.salahpour@utoronto.ca).
