# Supplementary material for: Tricyclic and tetracyclic antidepressants upregulate VMAT2 activity and rescue disease-causing VMAT2 variants
Source: Neuropsychopharmacology. 2024 Jul 26;49(11):1783–91. doi: 10.1038/s41386-024-01914-2 (PMC11399425; doi:10.1038/s41386-024-01914-2)
Supplement: Supplementary file 1 — Supplementary Materials [file 41386_2024_1914_MOESM1_ESM.docx]

**Supplementary Materials**

**Tricyclic and tetracyclic antidepressants upregulate VMAT2 activity and rescue disease-causing VMAT2 variants**

**Supplementary Methods**

FFN206 uptake assay

Epifluorescence microscopy

**Supplementary Tables**

Table S1. Reagent catalogue and supplier information

Table S2. Site-directed mutagenesis PCR primers used for constructing VMAT2 BVMTD variants.

Table S3. RT-qPCR primers used for quantitative assessment of gene expression.

**Supplementary Figures**

Figure S1. Example gating for BE(2)-M17 cell FFN206 uptake flow cytometry

Figure S2. Epifluorescent microscopy of FFN206 colocalization with VMAT2-mCherry.

Figure S3. 30-minute treatment of imipramine or miaserin on HEK293 cells with or without VMAT2.

Figure S4. Dose-response of 30-minute incubation with TBZ on VMAT2 activity.

Figure S5. 30-minute or 18-hour treatment of TBZ or haloperidol on HEK-VMAT2 cells.

Figure S6. Western blot analysis of VMAT2 deglycosylation.

Figure S7. Western blot analysis of 18-hour sustained imipramine or mianserin treatment on VMAT2 protein levels.

Figure S8. Western blot analysis of 18-hour sustained imipramine or mianserin treatment on YFP-DAT protein levels.

Figure S9. Western blot analysis of 18-hour sustained imipramine or mianserin treatment on SERT protein levels.

Figure S10. Western blot analysis of 18-hour sustained imipramine treatment on transiently transfected VMAT2 P237H protein levels.

Figure S11. Western blot analysis of 18-hour sustained imipramine treatment on transiently transfected VMAT2 P387L protein levels.

**Supplementary Methods**

**FFN206 uptake assay**

Black-bottomed 96-well plates were precoated overnight with 0.1mg/mL poly-D-lysine and UV sterilized before use. All incubations and washes were done at 37℃.

For the inhibition assay, HEK-VMAT2 cells were seeded at 50,000 cells/well on day 1. On day 2, the growth medium was aspirated and 90μL of drug-containing medium (1% DMSO, no G418) at varying concentrations were added to each well and incubated for 30min. After the incubation, 10μL of false fluorescent neurotransmitter (50μM FFN206, diluted in PBS) was added to each well, resulting in a 5μM final concentration of FFN206. Cells were incubated with FFN206 for 1hr. FFN206 uptake was terminated by aspirating the medium, and cells were washed twice with 200μL warm PBS (supplemented with 500μM MgCl_2_ and 900μM CaCl_2_), each with 5min incubation. Subsequently, 100μL warm PBS was added to each well and the plate was read with a Mithras LB 940 plate reader at excitation/emission filters 380nm/450nm. Treatment with 100 μM tetrabenazine (TBZ) in the vehicle was used to quantify VMAT2-independent FFN206 uptake and the signal was subtracted from each drug-treatment condition in data analysis.

For the upregulation assay, HEK-VMAT2 cells were seeded at 30,000 cells/well on day 1. We used different seeding density for inhibition assay (50,000 cells/well) and upregulation assay (30,000 cells/well) because the upregulation assay is one day longer than the inhibition assay. The cells can get overconfluent if seeded at 50,000 cells/well density on the first day. On day 2, the growth medium was aspirated and 100uL of medium containing drug or vehicle (1% DMSO, no G418) was added to each well and incubated for 18hrs. For dose-response experiments, the maximal dosage of compounds used was the highest dose that did not result in cellular toxicity. On day 3, the drug-containing medium was aspirated and washed 3 times with 200μL warm medium, each with 20min incubation. After the wash, 90μL medium containing 100μM TBZ (to measure VMAT2-independent uptake) diluted in 1% DMSO or vehicle (to measure VMAT2-specific uptake) were added to each well and incubated for 30min. After incubation, 10μL of 50μM FFN206 diluted in PBS was added to each well and incubated for 1hr. Afterwards the FFN206 uptake was terminated by 2 washes with 200μL warm PBS, each with 5min incubation. Subsequently, 100μL warm PBS was added to each well and the plate was read with a Mithras LB 940 plate reader. For each overnight treatment with 10μM compound, acute treatment with 100 μM TBZ in each condition on day 3 was used to define VMAT2-independent FFN206 uptake and the signal was subtracted from each drug-treated condition during data analysis. For dose-response of overnight compound treatment, acute treatment with 100 μM TBZ in the vehicle on day 3 was used to define VMAT2-independent FFN206 uptake and the signal was subtracted from each drug-treated condition during data analysis.

**Epifluorescence microscopy**

FFN206 uptake was performed as previously described [21]. Briefly, HEK-mCherry-VMAT2 cells were seeded in a clear 6-well plate at 250,000 cells/well density. 24hrs later, culture media was removed and cells were pre-incubated with DMSO or 10μM TBZ (to measure VMAT2-independent uptake) for 30min in DMEM. After 30min, FFN206 prepared in DMEM was added to a final concentration of 5μM and incubated for 1hr. Afterwards, media was removed and replaced with DMEM containing 1:25 trypan blue to reduce background fluorescence and cells were imaged on an Olympus APEX100 microscope at 40x magnification using a DAPI filter to visualize FFN206 and mCherry/TexasRed filter to visualize mCherry-tagged VMAT2.

**Table S1. Reagent catalogue and supplier information**

| **Categories** | **Reagent (catalogue)** | **Supplier** |
| --- | --- | --- |
| Cell lines | HEK293 (CRL-1573) | ATCC |
|  | HEK293T (CRL-3216) | ATCC |
|  | BE(2)-M17 (CRL-2267) | ATCC |
| Reagents | Penicillin and streptomycin solution, 100X (PST999) | Bioshop |
|  | G418 sulfate powder (GEN418.5) | Bioshop |
|  | CELLSTAR® 96 well Microplates, black plate with black bottom (655086) | Greiner Bio-One |
|  | Dulbecco′s Phosphate Buffered Saline, with MgCl2 and CaCl2 (D8662) | Sigma-Aldrich |
|  | 6-well plate, clear (3516) | Corning |
|  | Trypsin-EDTA (0.25%) (25200056) | Gibco |
|  | Pierce™ BCA Protein Assay Kits (23225) | ThermoFisher Scientific |
|  | Endo H (P0702S) | New England Biolabs, Inc. |
|  | PNGase F (P0704S) | New England Biolabs, Inc. |
|  | NuPAGE™ 4 to 12% Bis-Tris Mini Protein Gel (NP0322BOX) | Invitrogen |
|  | Revert™ 700 Total Protein Stain | LI-COR, Inc. |
|  | rabbit-anti-GFP antibody (A11122) | Invitrogen |
|  | IRDye® 800CW Goat anti-Rabbit IgG (926-32211) | LI-COR, Inc. |
|  | Lipofectamine^TM^ 3000 | Invitrogen |
|  | TRIzol Reagent | Invitrogen |
|  | SuperScript™ IV VILO™ Master Mix with ezDNase™ Enzyme | Invitrogen |
|  | PowerUp™ SYBR™ Green Master Mix | Applied Biosystems |
| Chemicals | FFN206 dihydrochloride (5043) | Tocris Bioscience |
|  | Tetrabenazine (T284000) | Toronto Research Chemicals |
|  | Reserpine (R144600) | Toronto Research Chemicals |
|  | Mianserin hydrochloride (M341500) | Toronto Research Chemicals |
|  | Amitriptyline hydrochloride (A633350) | Toronto Research Chemicals |
|  | Amoxapine (A634230) | Toronto Research Chemicals |
|  | Desipramine hydrochloride (D290050) | Toronto Research Chemicals |
|  | Doxepin hydrochloride (D550000) | Toronto Research Chemicals |
|  | Imipramine hydrochloride (I465980) | Toronto Research Chemicals |
|  | Nortriptyline hydrochloride (N837000) | Toronto Research Chemicals |
|  | Trimipramine maleate salt (T799000) | Toronto Research Chemicals |
|  | Protriptyline hydrochloride (P838875) | Toronto Research Chemicals |

**Table S2. Site-directed mutagenesis PCR primers used for constructing VMAT2 BVMTD variants.**

| **Construct** | **Mutation** | **Forward primer (5' to 3')** | **Reverse primer (5' to 3')** |
| --- | --- | --- | --- |
| pcDNA3.1-hVMAT2-P42L | P42L | gctcactgtcgtggtcctcatcatcccaagttatc | gataacttgggatgatgaggaccacgacagtgagc |
| pcDNA3.1-hVMAT2-I43F | I43F | cactgtcgtggtccccttcatcccaagttatct | agataacttgggatgaaggggaccacgacagtg |
| pcDNA3.1-hVMAT2-P237H | P237H | ttagtgggcccccacttcgggagtgtg | cacactcccgaagtgggggcccactaa |
| pcDNA3.1-hVMAT2-A309V | A309V | caaacatgggcatcgtcatgctggagccagc | gctggctccagcatgacgatgcccatgtttg |
| pcDNA3.1-hVMAT2-P316A | P316A | ggagccagccctggccatctggatgat | atcatccagatggccagggctggctcc |
| pcDNA3.1-hVMAT2-A333D | A333D | gcagctgggcgttgacttcttgccagcta | tagctggcaagaagtcaacgcccagctgc |
| pcDNA3.1-hVMAT2-P387L | P387L | catttatggactcatagctctgaactttggagttggttttgc | gcaaaaccaactccaaagttcggagctatgagtccataaatg |
| pcDNA3.1-hVMAT2-Y418C | Y418C | gcggcacgtgtccgtctgtgggagtgt | acactcccacagacggacacgtgccgc |
| pcDNA3.1-hVMAT2-G436S | G436S | tttgtatggggtatgctataagtccttctgctggtg | caccagcagaaggacttatagcataccccatacaaa |

**Table S3. RT-qPCR primers used for quantitative assessment of gene expression.**

| **Gene** | **Forward (5' to 3')** | **Reverse (5' to 3')** | **Sequence** | **Efficiency (90-110%)** | **Ct** |
| --- | --- | --- | --- | --- | --- |
| hGAPDH | GTCTCCTCTGACTTCAACAGCG | ACCACCCTGTTGCTGTAGCCAA | NM_001256799.3 | 96.18 | 20.17 |
| mVMAT2 | CCTCTTACGACCTTGCTGAAGG | GCTGCCACTTTCGGGAACACAT | BC078449.1 | 99.87 | 22.61 |
| hVMAT2 | GCTATGCCTTCCTGCTGATTGC | CCAAGGCGATTCCCATGACGTT | NM_003054.6 | 101.39 | 27.71 |
| hVMAT1 | AGGTTTCTTGGAGGAAGAGATTAC | ATCCAATCCTGTTGGTGAGAG | NM_003053.4 | 105.03 | 27.48 |
| hDAT | CCTCAACGACACTTTTGGGACC | AGTAGAGCAGCACGATGACCAG | NM_001044.5 | 109.23 | 22.41 |
| hSERT | TCACAGTGCTCGGTTACATGGC | GAAAGTGGACGCTGGCATGTTG | NM_001045.6 | 94.43 | 19.10 |
| hNET | CAGGTTCAGCAACGACATCCAG | GTCGTAGGTGAGTGGCTTGAAG | NM_001043.3 | 108.09 | 23.18 |

**GAPDH:** Glyceraldehyde 3-phosphate dehydrogenase; **VMAT:** Vesicular Monoamine Transporter; **DAT:** dopamine transporter; **SERT:** Serotonin transporter; **NET:** Norepinephrine transporter.

**
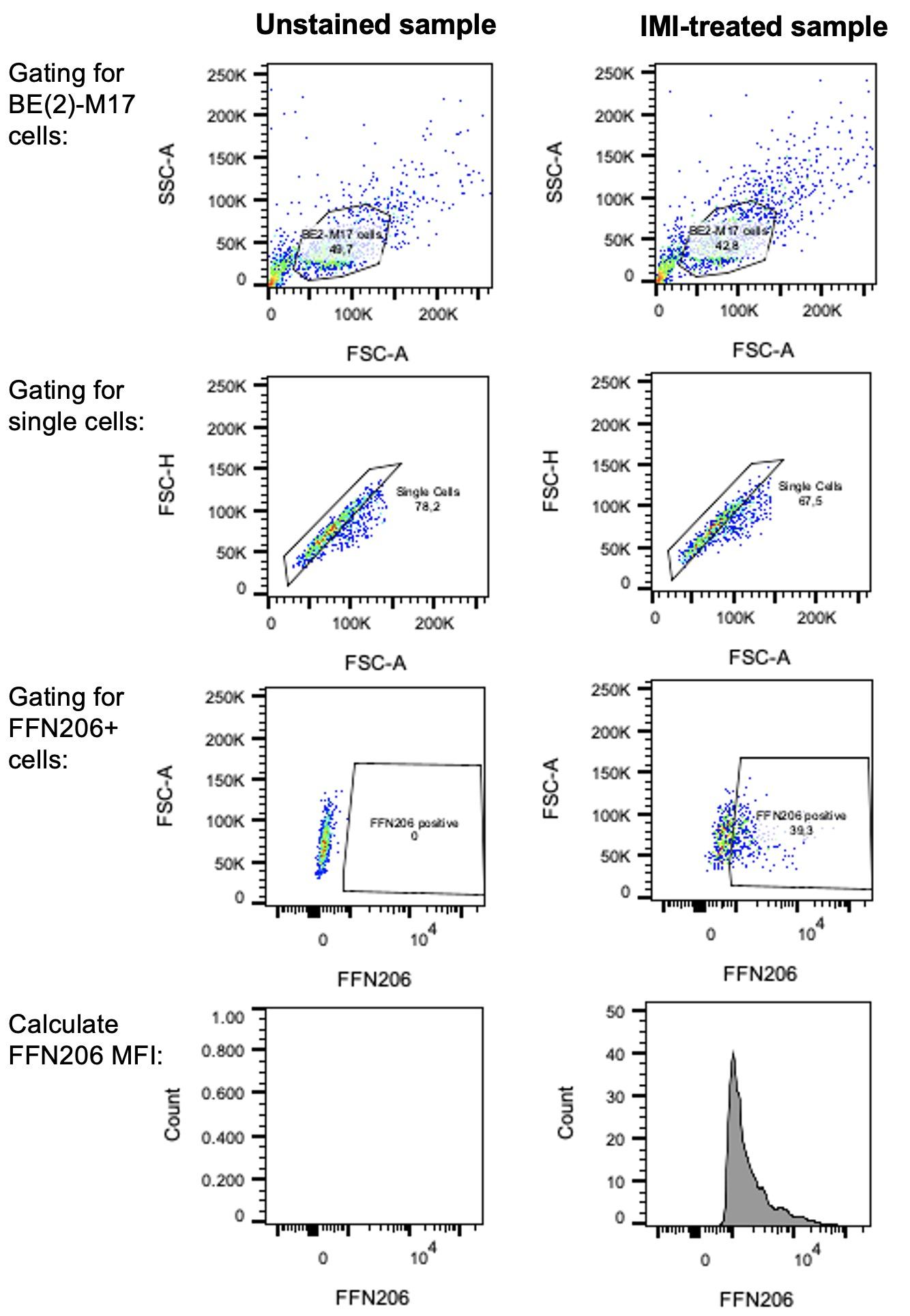
**

**Figure S1. Example gating for BE(2)-M17 cell FFN206 uptake flow cytometry.** Gatings: BE(2)-M17 cells/single cells/FFN206 positive cells. Mean fluorescence intensity (MFI) was quantified for FFN206 positive cells. Left: unstained sample (no FFN206); right: imipramine-treated sample with FFN206.


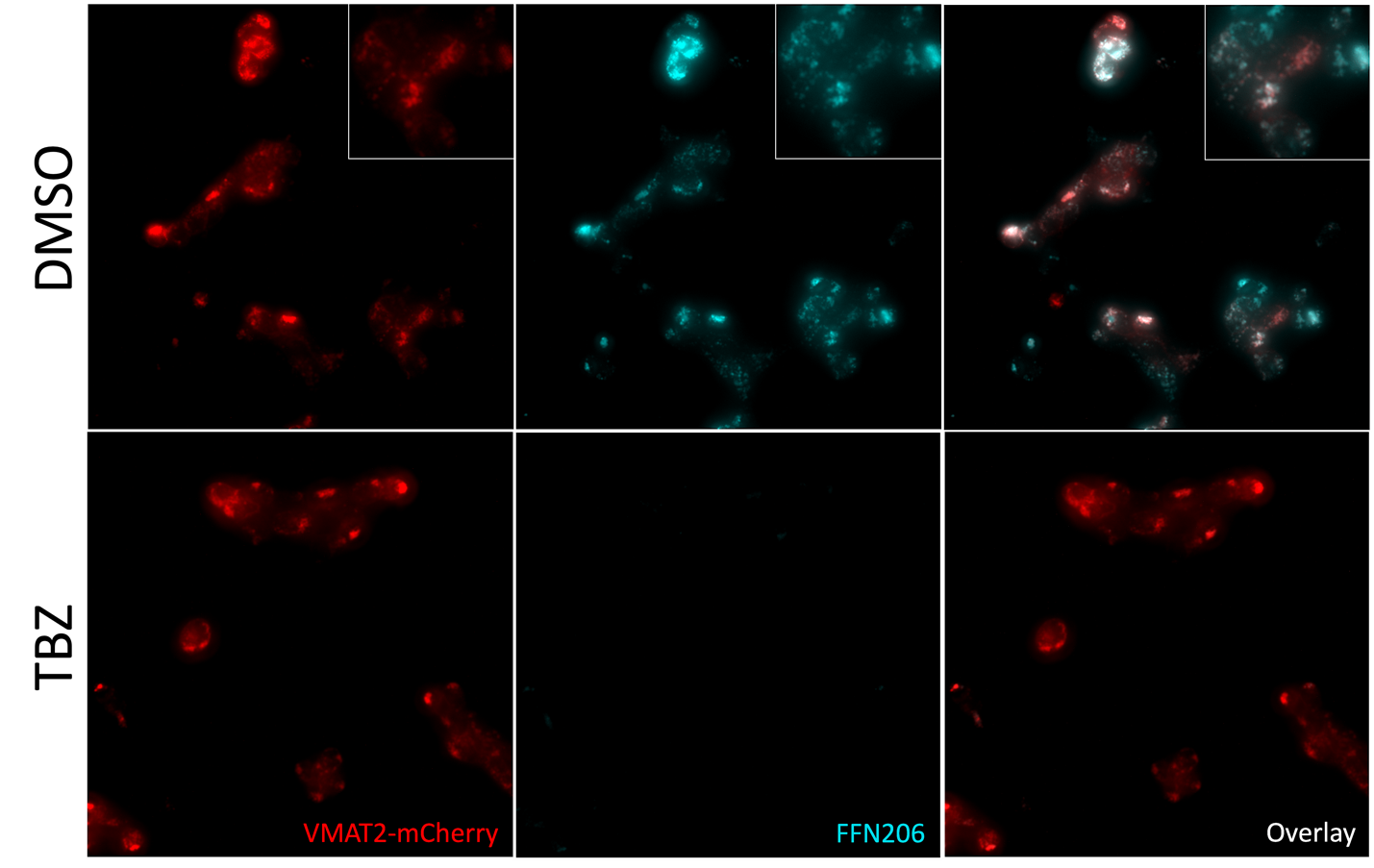


**Figure S2. Epifluorescent microscopy of VMAT2-mCherry and FFN206.** Red: VMAT2-mCherry; Cyan: FFN206; White: Overlay of VMAT2-mCherry and FFN206. VMAT2 and FFN206 colocalize in intracellular compartments.

**
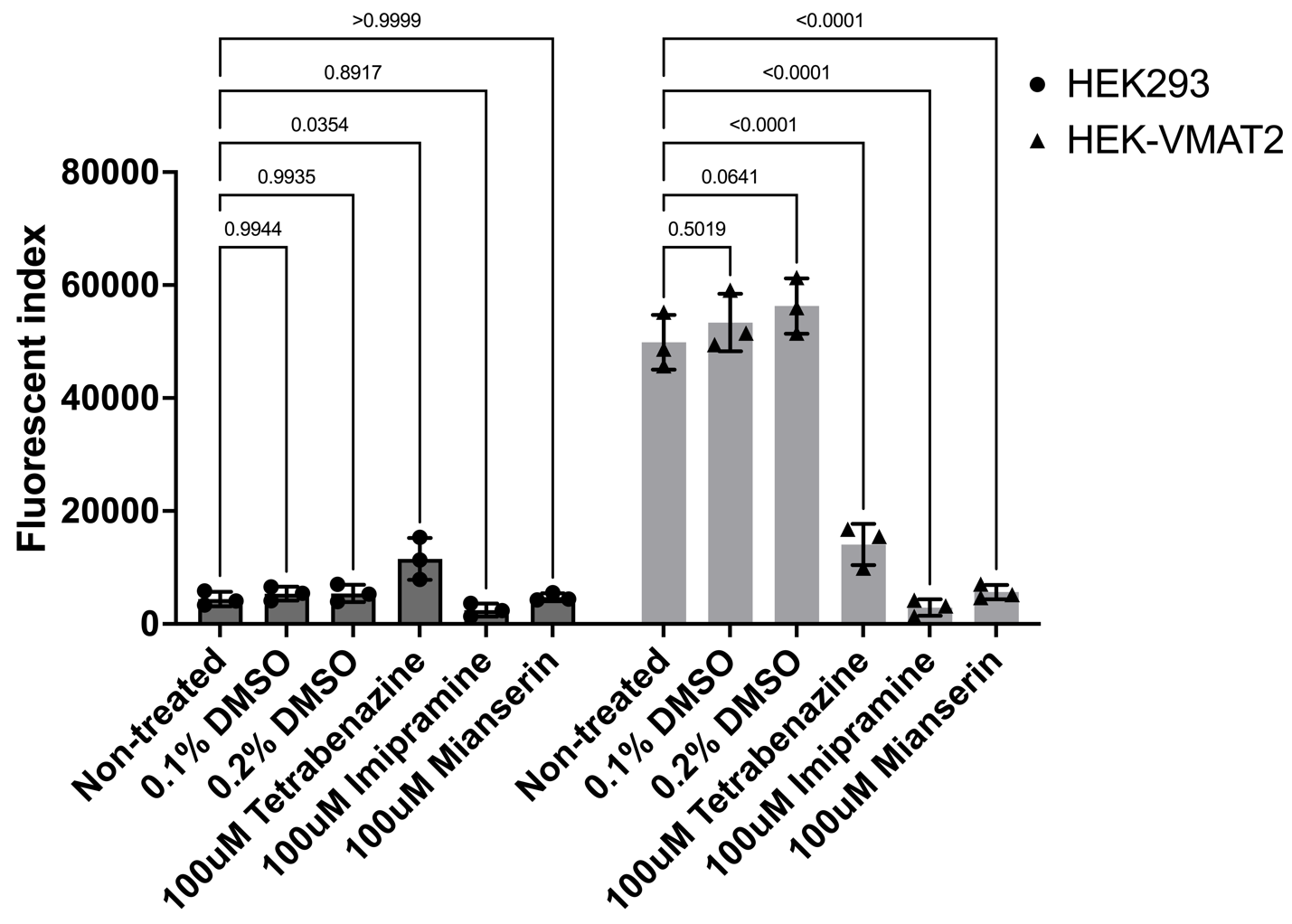
**

**Figure S3. 30-minute treatment of imipramine or mianserin on HEK293 cells with (HEK-VMAT2) or without VMAT2 (HEK293) (n=3).** Two-way ANOVA was performed followed by Dunnett’s test pairwise comparison against not-treated condition.

**
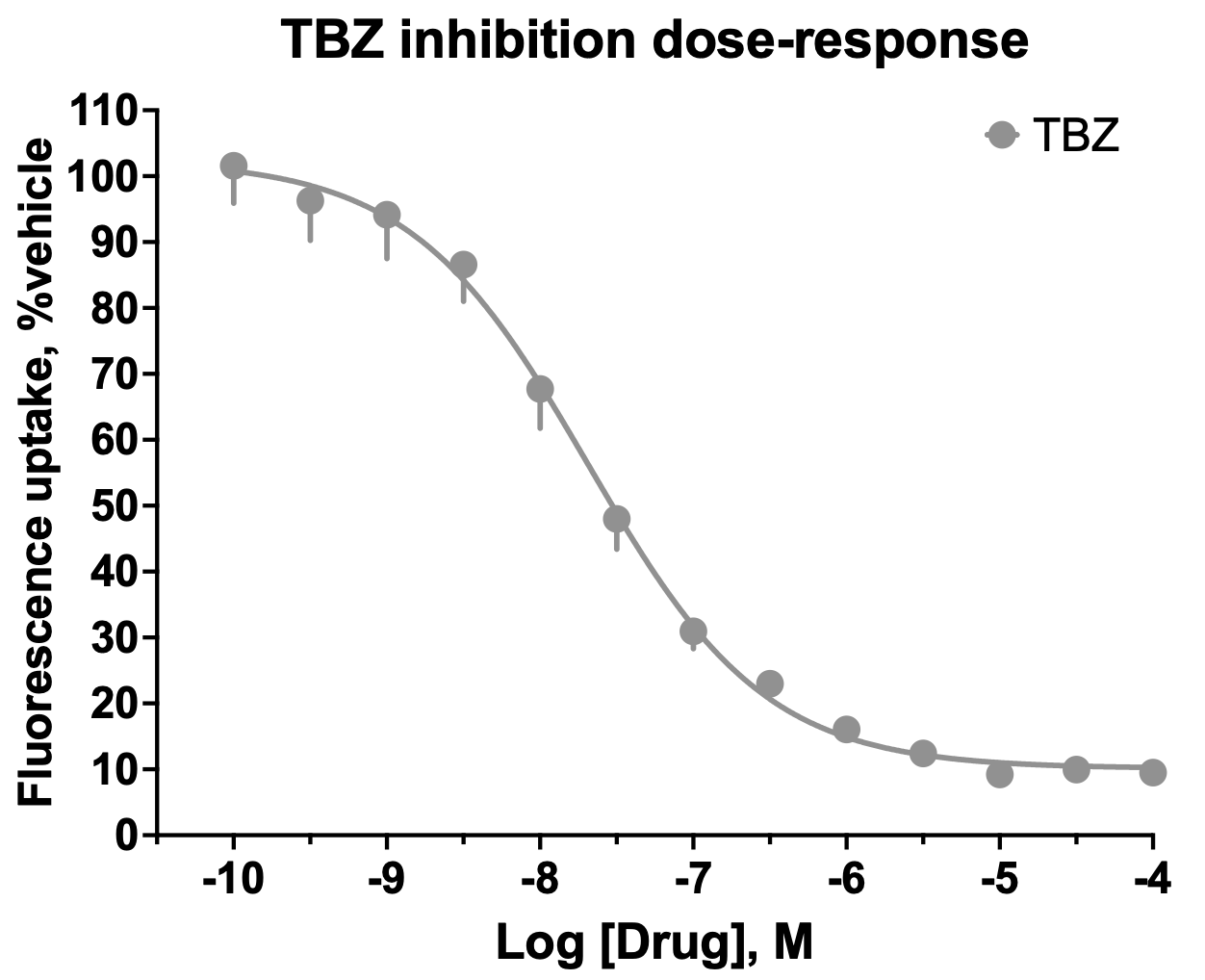
**

**Figure S4. Dose-response of 30-minute incubation with TBZ on VMAT2 activity in HEK-VMAT2 cells (n = 10).**

**
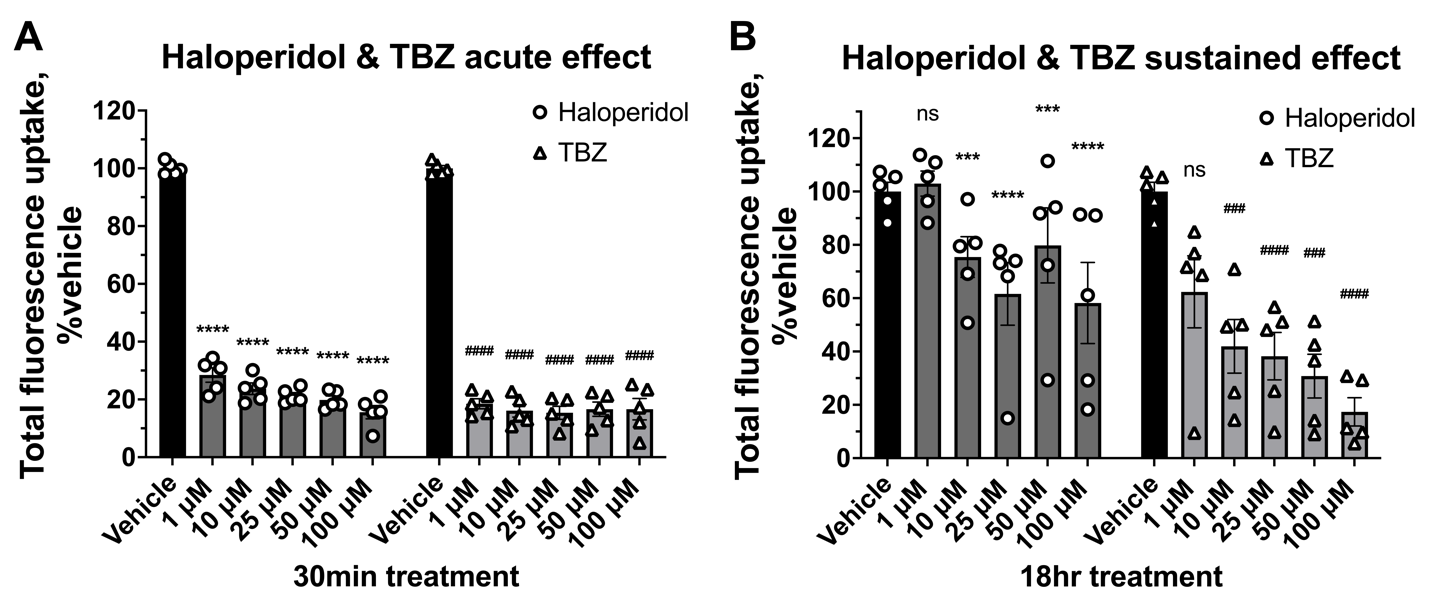
**

**Figure S5. 30-minute or 18-hour incubation with TBZ or haloperidol on HEK-VMAT2 cells (n = 5). (A)** 30-minute incubation with increasing concentrations of TBZ or haloperidol on HEK-VMAT2 cells (n = 5). **(B)** 18-hour incubation with increasing concentrations of TBZ or haloperidol on HEK-VMAT2 cells (n = 5). Two-way ANOVA was performed followed by Dunnett’s test pairwise comparison against vehicle condition. P > 0.05 (ns), ≤ 0.05 (*), ≤ 0.01 (**), ≤ 0.001 (***), ≤ 0.0001 (****). All treatments were normalized to vehicle (100%).

**
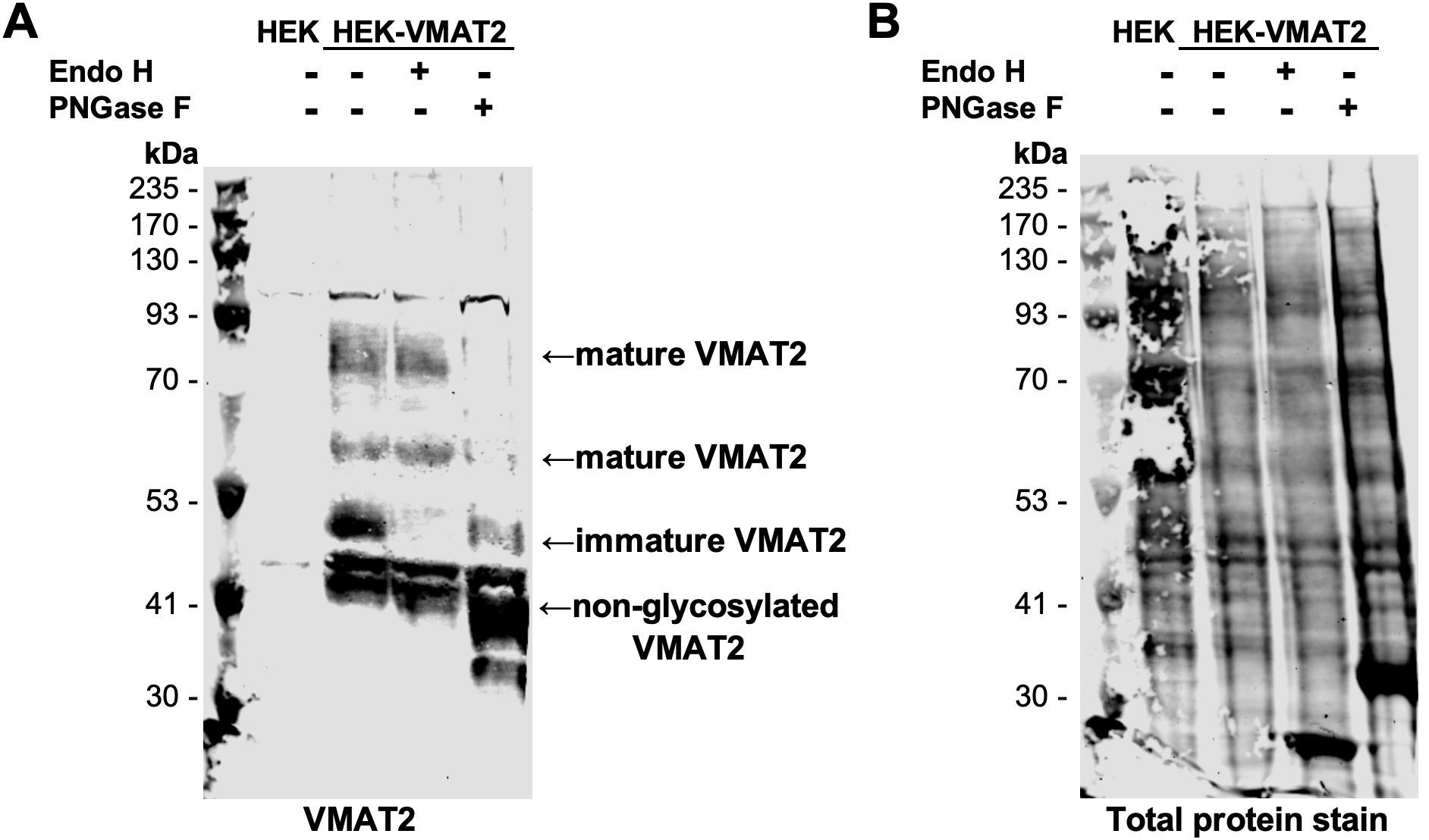
 Figure S6. Western blot analysis of VMAT2 deglycosylation. (A)** Western blot of endoglycosidase H (EndoH) and peptide:N-glycosidase F (PNGase F) digested VMAT2 protein revealing different trafficking stages of the VMAT2 protein. **(B)** Total protein stain of blot A as a loading control.

**
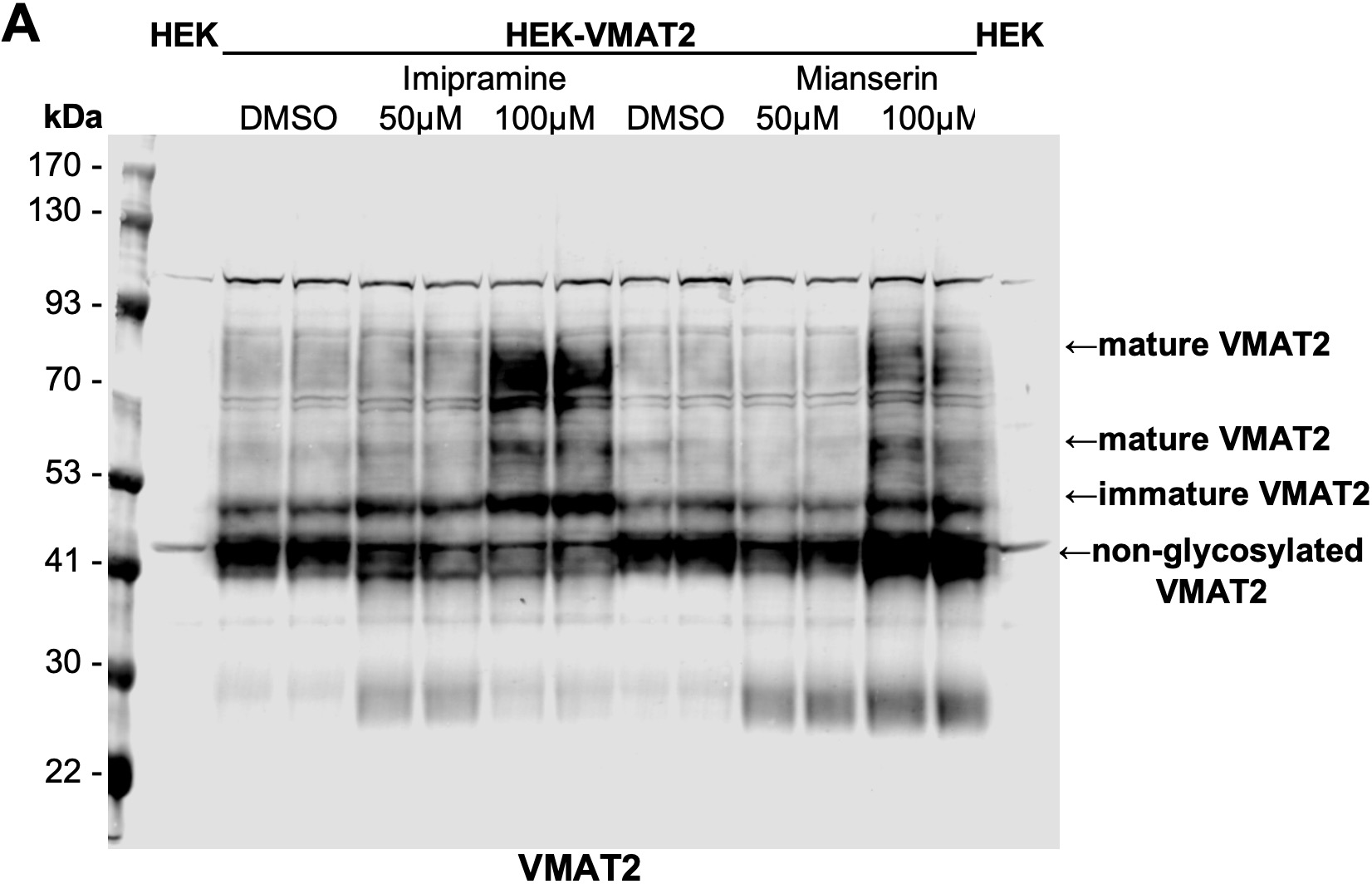
** **
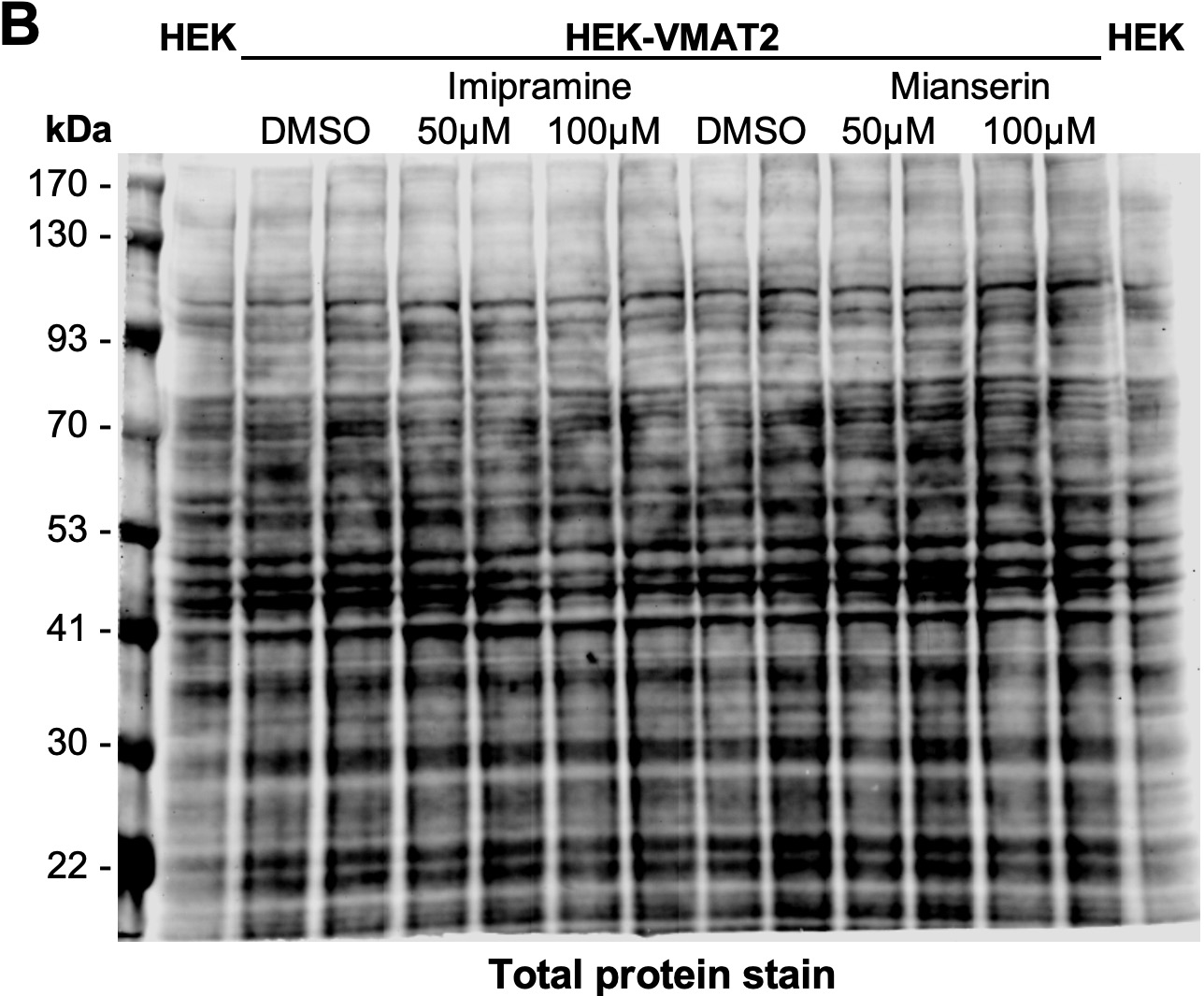
**

**Figure S7. Western blot analysis of 18-hour sustained imipramine or mianserin treatment on VMAT2 protein levels. (A)** Representative western blot of 18-hour imipramine or mianserin incubation on VMAT2 protein in HEK-VMAT2 cells. Duplicate wells (n=1) displayed. **(B)** Total protein stain of blot A as a loading control.

**
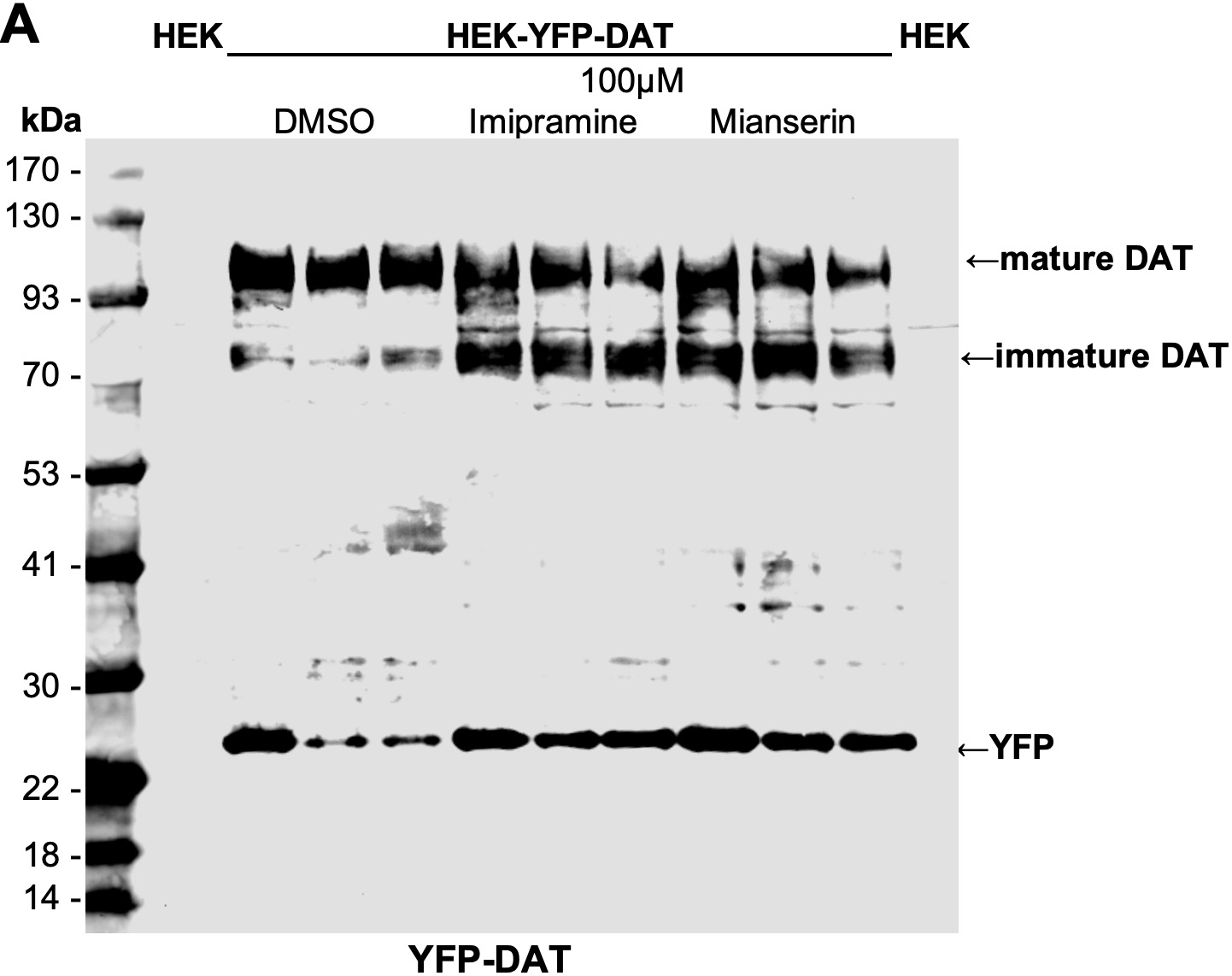
** **
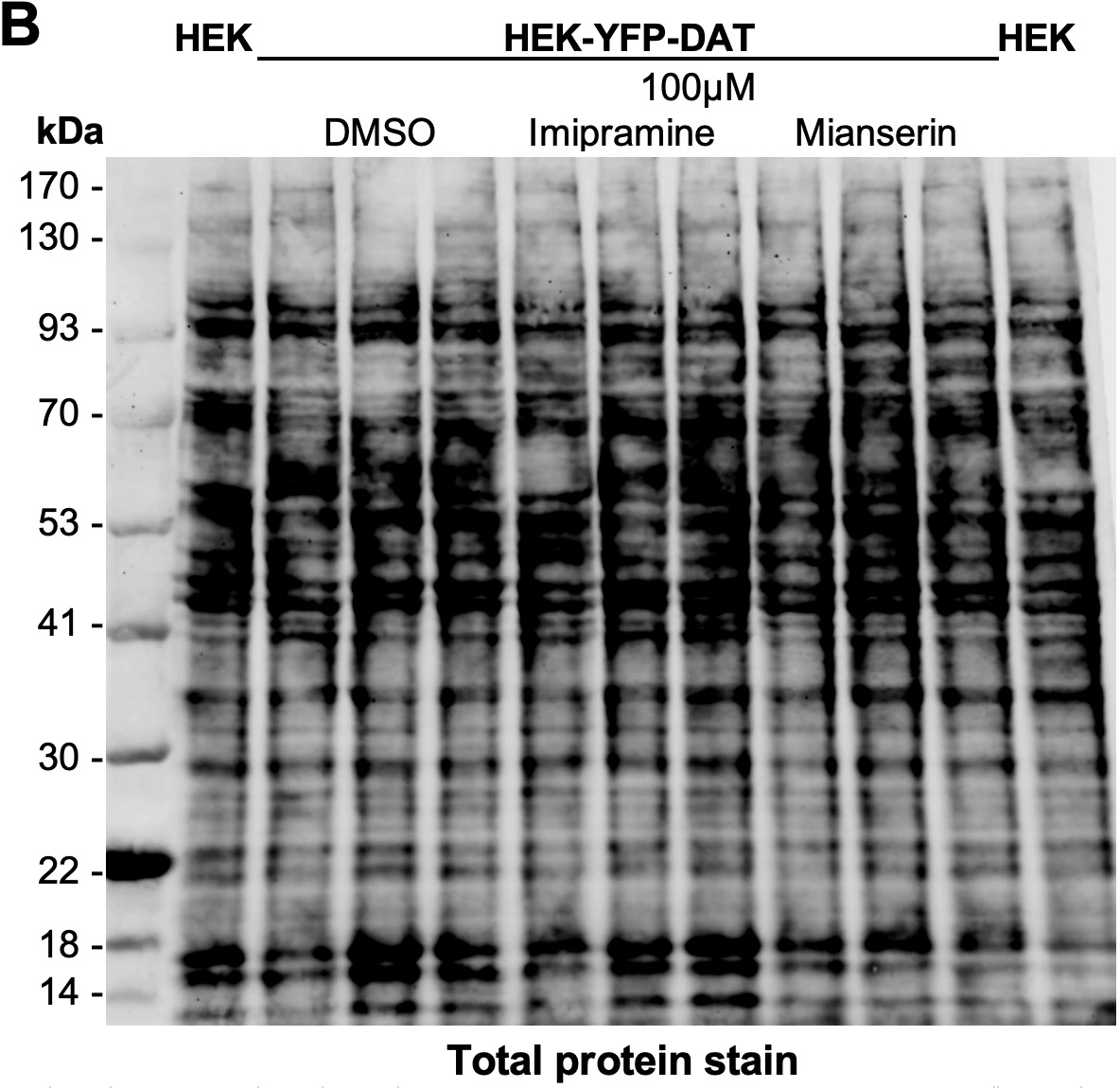
**

**Figure S8. Western blot analysis of 18-hour sustained imipramine or mianserin treatment on YFP-DAT protein levels. (A)** Western blot of 18-hour 100 μM imipramine and mianserin incubation on DAT protein in HEK-YFP-DAT cells (n = 3, each well represents an independent experiment). **(B)** Total protein stain of blot A as a loading control.

**
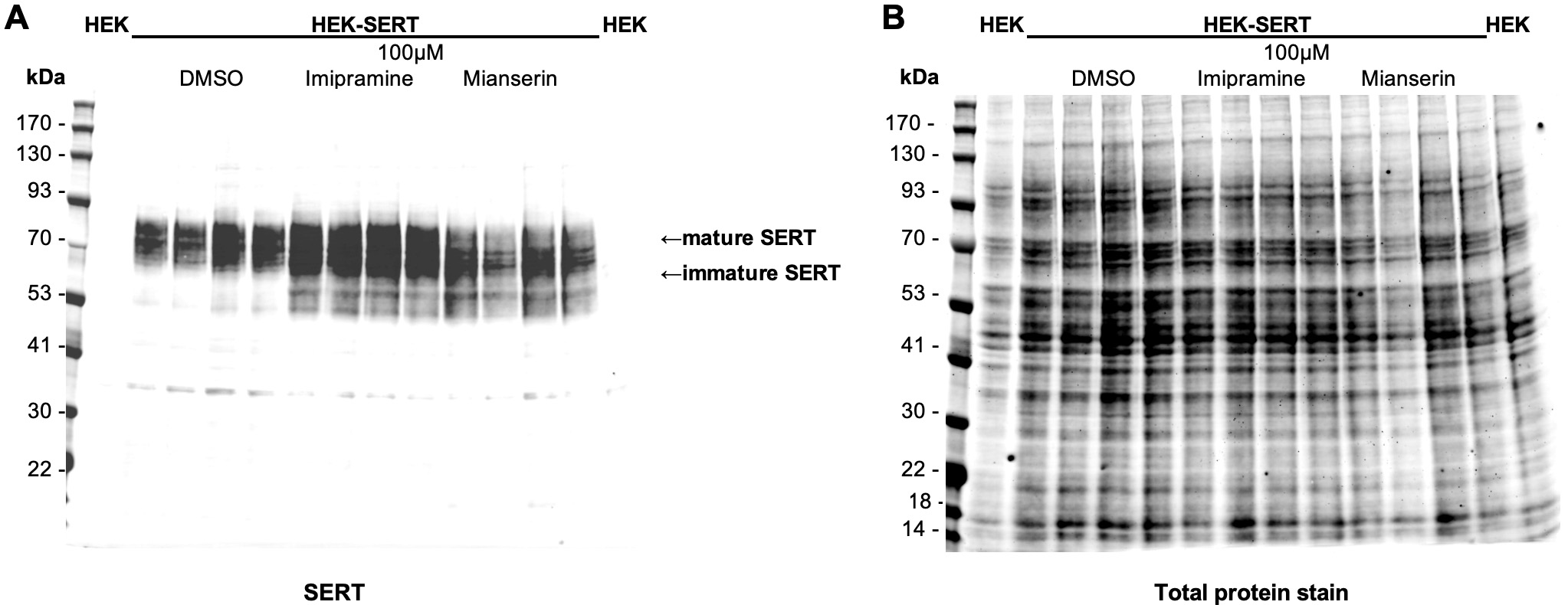
Figure S9. Western blot analysis of 18-hour sustained imipramine or mianserin treatment on SERT protein levels. (A)** Western blot of 18-hour 100 μM imipramine and mianserin incubation on SERT protein in HEK-SERT cells (n = 4, each well represents an independent experiment). **(B)** Total protein stain of blot A as a loading control.


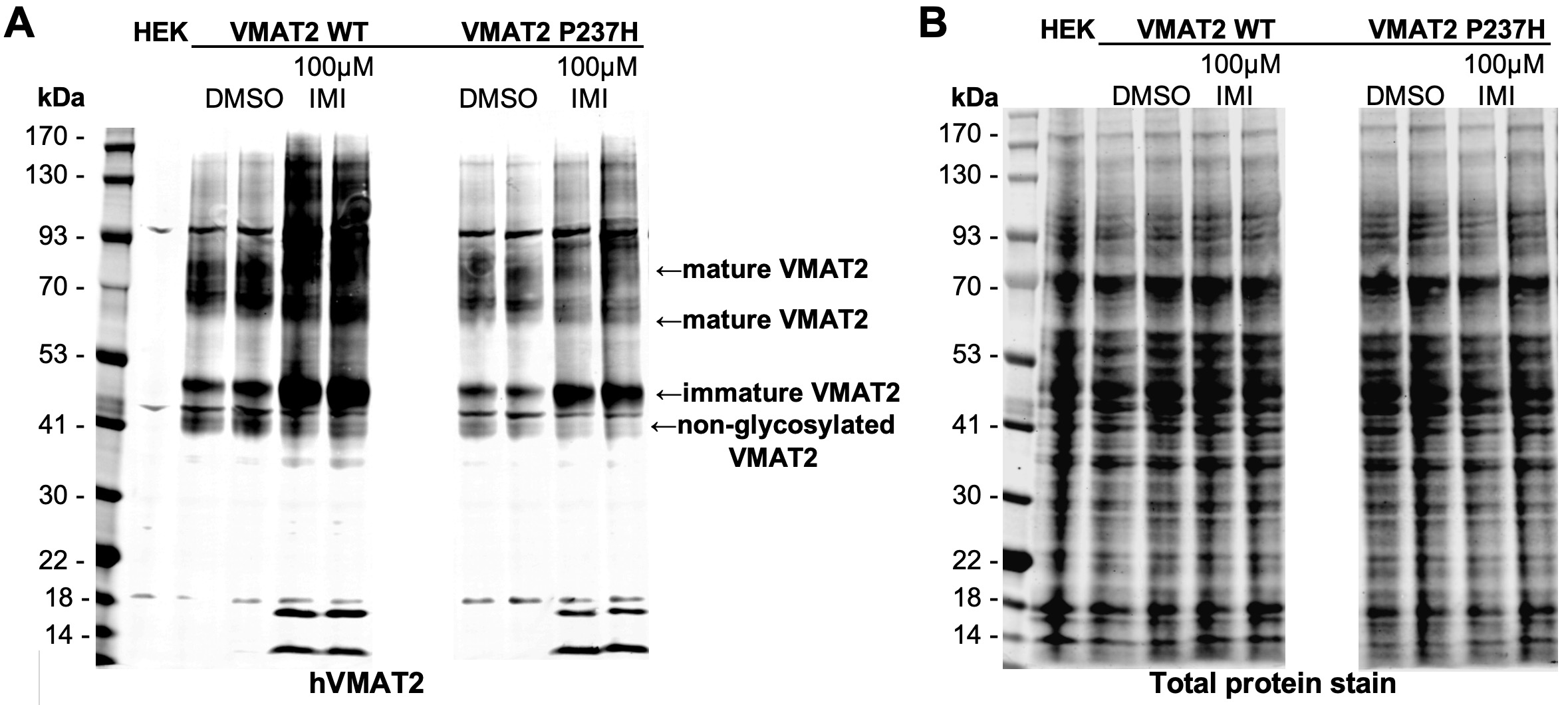


**Figure S10. Western blot analysis of 18-hour sustained imipramine on VMAT2 P237H protein levels. (A)** Representative western blot of 18-hour 100 μM imipramine incubation on WT or P237H protein in HEK293T cells (n=4 in total, n = 2 displayed, each well represents an independent experiment). **(B)** Total protein stain of blot A as a loading control. VMAT2 WT and P237H were ran on the same gel and cropped to remove unrelated lanes.


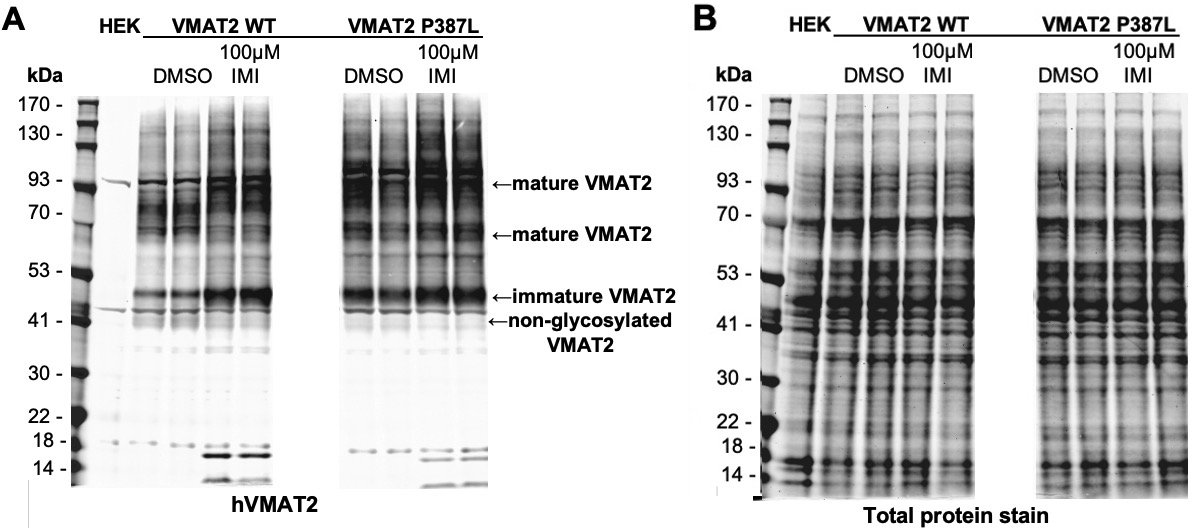


**Figure S11. Western blot analysis of 18-hour sustained imipramine on VMAT2 P387L protein levels. (A)** Representative western blot of 18-hour 100 μM imipramine incubation on WT or P387L protein in HEK293T cells (n=4 in total, n = 2 displayed, each well represents an independent experiment). **(B)** Total protein stain of blot A as a loading control. VMAT2 WT and P387L were ran on the same gel and cropped to remove unrelated lanes.
